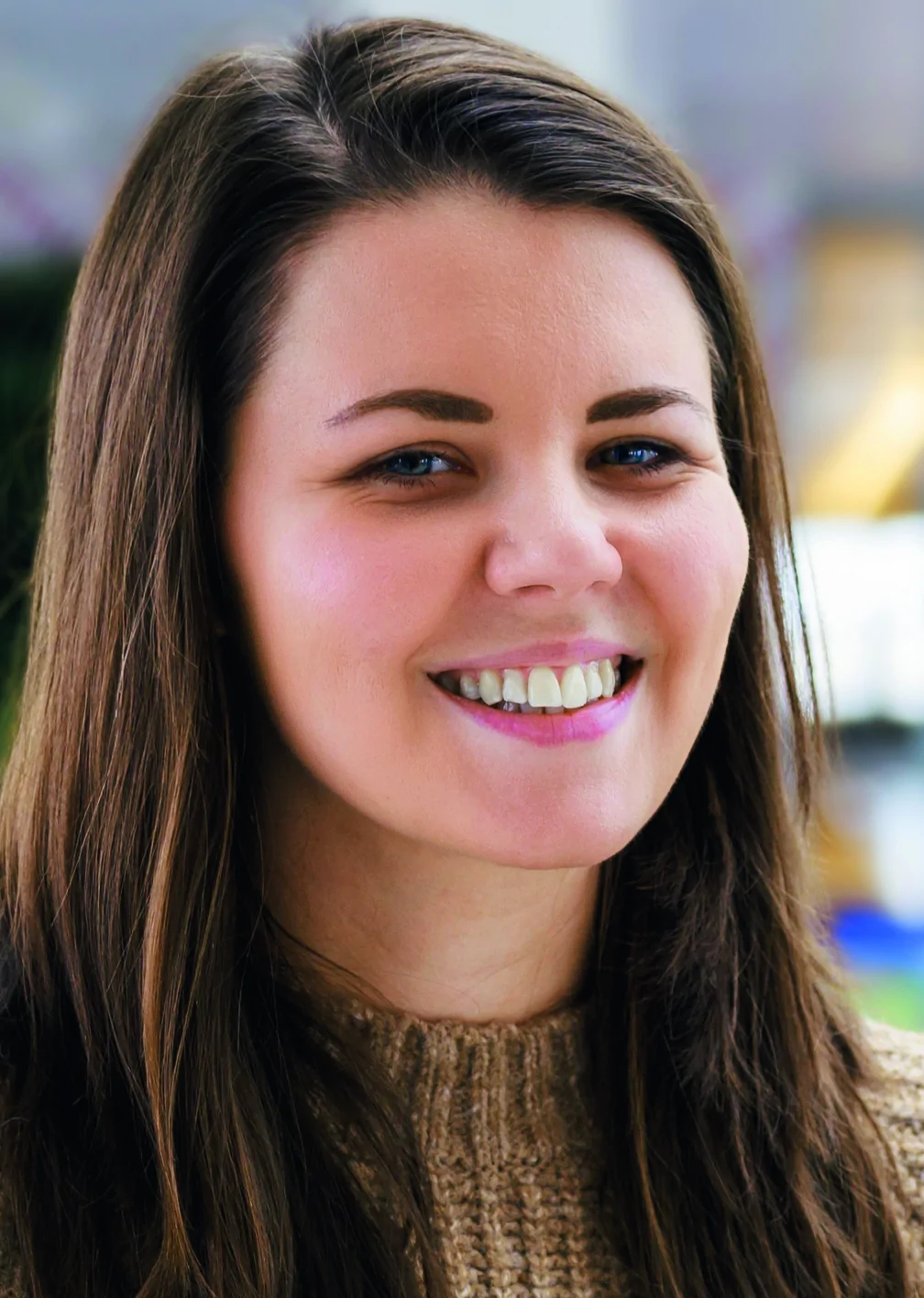# Ideas Behind the Tryptophan‐Mediated Petasis Reaction (TMPR) Concept for Peptide Stapling

**DOI:** 10.1002/cmdc.202400148

**Published:** 2024-06-20

**Authors:** Sona Krajcovicova

**Affiliations:** ^1^ Yusuf Hamied Department of Chemistry University of Cambridge Lensfield Road CB2 1EW Cambridge United Kingdom; ^2^ Department of Organic Chemistry Faculty of Science Palacky University in Olomouc Tr. 17. Listopadu 12 77900 Olomouc Czech Republic

**Keywords:** tryptophan, Petasis reaction, multicomponent reactions, peptide stapling, peptide labelling

## Abstract

This *Concept* short review offers an insightful analysis of pivotal research papers and explores the key synthetic ideas behind the intersection of two realms in peptide chemistry: using tryptophan and Petasis multicomponent reactions for macrocyclisation and labelling of peptides. The recently published tryptophan‐mediated Petasis reaction (TMPR) concept represents a critical junction between these two worlds, highlighting how combining such methodologies leads to more effective and versatile synthetic strategies, setting a potentially new direction for future research in the field of peptide‐drug conjugates.

## Graphical abstract

This *Concept* review explores the pioneering role of the Petasis multicomponent reaction in peptide stapling and labelling and the use of the tryptophan indole core in peptide functionalisation. TMPR bridges the Petasis reaction and tryptophan integration, offering a novel approach to synthetic strategies in peptides. It focuses on key research papers that guided and helped to develop the idea behind this concept.

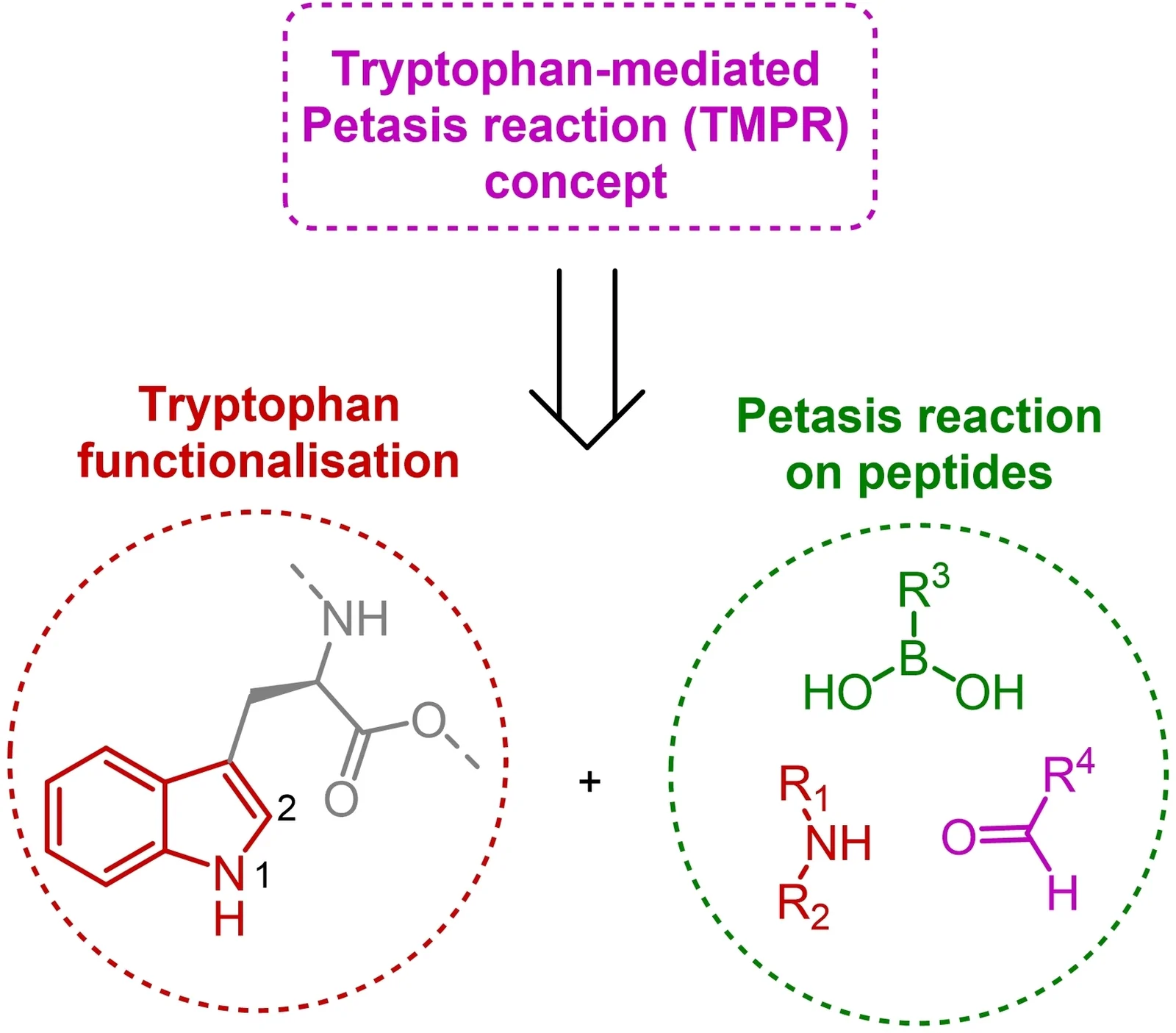

## Introduction

The advancements in peptide‐based therapeutics, in order to improve the often‐poor pharmacokinetic limitations of peptides, have seen a significant growth in the last decade. One of the driving forces for these advancements was peptide macrocyclisation, also known as a peptide stapling.

**Figure 1:**
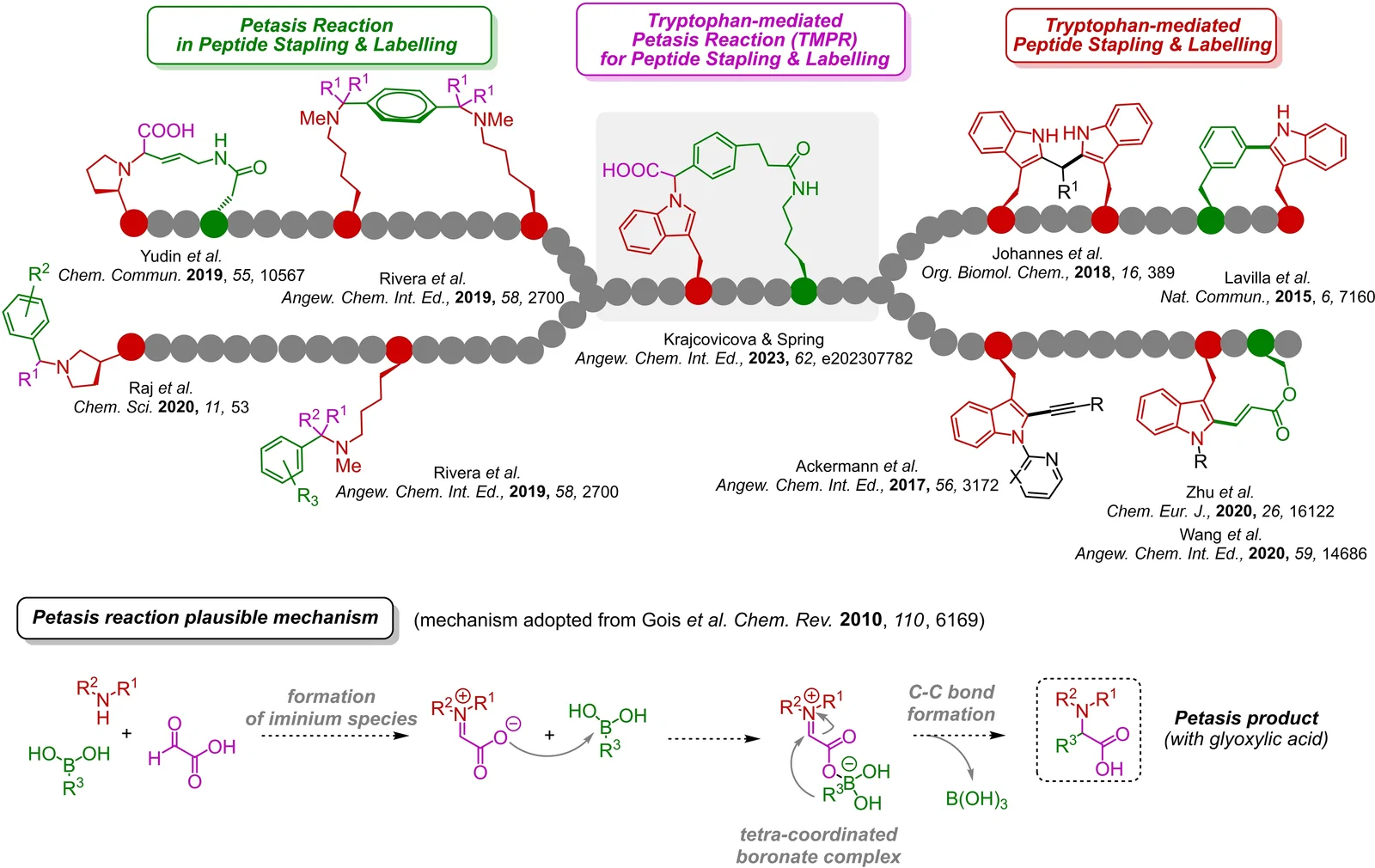
Intersection of two realms.

Peptide stapling, which is usually a side chain‐to‐side chain tethering of two amino acid residues separated along the sequence, enhances the stabilisation of α‐helical structures, leading to peptides that are not only more stable and cell‐permeable but also exhibit increased binding affinity.^[1]^ This approach has shown considerable promise in targeting protein‐protein interactions related to a variety of diseases, including cancer and neurological disorders. Macrocyclisation is thus generally regarded as the most efficient method for imposing conformational constraints in short‐ and medium‐length peptides.^[1]^ Commonly used amino acids in this context include lysine, aspartic acid, cysteine, or their unnatural modifications like azides and alkynes. Tryptophan, despite its diverse reactivity potential stemming from its versatile heterocyclic indole core and lower abundance in natural proteins, was not typically the first choice considered. However, in recent years, the increasing focus on discovering novel and chemoselective methods for peptide stapling has established it as a promising option for various synthetic transformations, both transition metal‐catalysed[^2^, ^3^, ^4^, ^5^, ^6^, ^7^, ^8^, ^9^] as well as metal‐free[^10^, ^11^, ^12^, ^13^, ^14^] (Figure 1).

In parallel, multicomponent reaction approaches represent a powerful method for accessing biologically active molecules and have gained significant attention over the last couple of years.[^1^, ^15^, ^16^, ^17^] The ability to produce a wide range of natural‐product‐like compounds highlights its importance in the development of new therapeutics and biological probes, underlining the ongoing evolution and significance of these methodologies in medicinal chemistry. Although the importance of multicomponent reactions in macrocyclisation chemistries is well acknowledged, their effective integration into peptide stapling techniques has only been realised recently, with significant exploration of the Ugi four‐component reaction.^[1]^ Other multicomponent reactions, such as the Petasis reaction (Figure 1; *Plausible mechanism*), gained significantly less attention, despite them being similarly powerful synthetic transformations.

The recent interest in other multicomponent options has made the Petasis reaction (the transformation of a boronic acid, an amine, and a carbonyl derivative), a promising basis for the development of innovative methods for its application.[^15^, ^18^, ^19^]

Building on these methodologies, the recently published TMPR concept^[10]^ represents a pioneering intersection between the multicomponent Petasis reaction and the unique properties of tryptophan in peptide stapling. This approach not only improves peptide stability but also allows for innovative late‐stage functionalisation, marking a significant advancement in the future of peptide‐based therapeutics.

This *Concept* short review aims to discuss the quite recent but pioneering role of the Petasis multicomponent reaction in peptide stapling and the integration of the tryptophan indole core into the macrocyclisation of peptides. TMPR serves as a critical junction between these two realms (Figure 1), showcasing how a combination of such methodologies could lead to more effective and versatile synthetic strategies, setting a new direction for future research in the field of peptide therapeutics. The aim of this short *Concept* review is hence to discuss only the key ideas that helped develop the TMPR concept and thus it does not aim to thoroughly review all aspects of Petasis multicomponent reactions and tryptophan in peptide stapling. To gain such information, the reader is advised to focus on the other previously published and more comprehensive reviews.[^1^, ^16^, ^20^, ^21^, ^22^, ^23^]

## Petasis Reaction in Stapling, Labelling and Macrocyclisation of Peptides

Recent advancements in peptide chemistry have shown the diverse applications of the Petasis reaction (PR) in enhancing peptide‐based research. Several techniques have been developed for precise peptide labelling and stapling, allowing for the addition of various functional groups to increase molecular complexity.^[17]^ Additionally, novel methods for selective bioconjugation and the assembly of conformationally stable peptide macrocycles have been introduced, significantly broadening the scope and potential of peptide therapeutics and bioactive compound development.^[18]^

**Figure 2:**
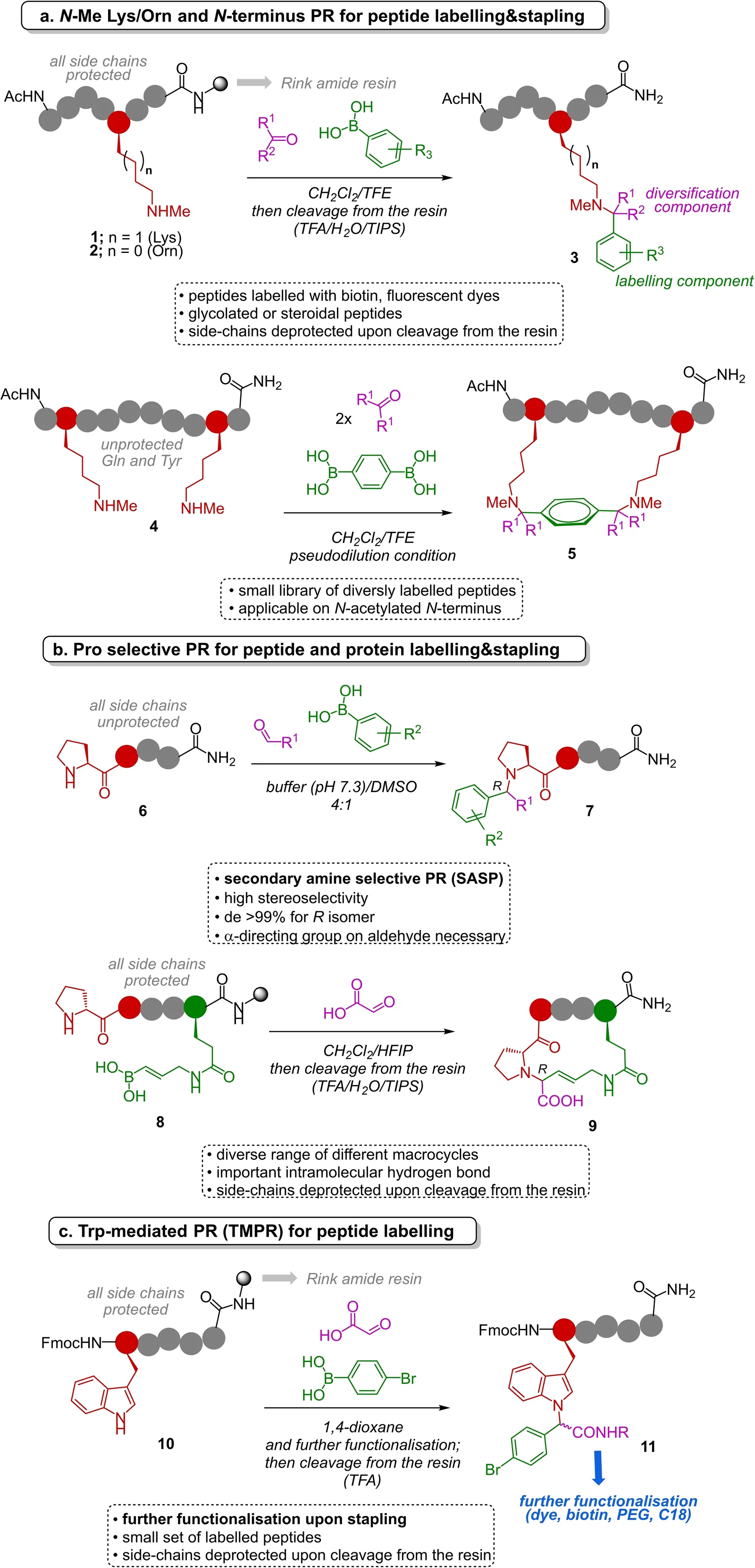
Initial applications of Petasis reaction in peptide macrocycliclisation and labelling.[^10^, ^17^, ^18^, ^19^]

Pioneering work from Rivera *et al*.^[17]^ introduces an innovative application of the PR for peptide stapling and labelling. Their work demonstrates the solid‐phase derivatisation of peptides at the *N*‐terminus and *via* the *N*‐methyl lysine/ornithine side‐chains (**1** or **2**, Figure 2a), allowing for the addition of various functional groups without altering the peptide charge. Notably, their research highlights the simultaneous incorporation of aryl and vinyl substituents, fluorescent or affinity tags, and oxo components, enhancing peptide complexity (**3** and **5**, Figure 2a). The authors also successfully employ multicomponent stapling in solution, linking side chains with aryl tethers and introducing hydroxy carbonyl moieties as exocyclic fragments. These findings underscore the potential of the PR potential in peptide drug discovery and chemical biology, demonstrating its efficacy and versatility (Figure 2a).

Another application of secondary aliphatic amines, in this case *N*‐terminal proline in the PR for labelling of peptides and proteins, has been introduced by Raj *et al*.^[18]^ (Figure 2b). Their method allows for the synthesis of functionalised secondary amines under physiological conditions, exhibiting remarkable chemoselectivity and stereoselectivity (**7**; >99 % *de*). This advancement encompasses the formulation of a selective bioconjugation process for peptides and proteins, which remains effective across a range of pH conditions and is versatile enough to accommodate various arylboronic acids. Its expanded substrate scope, including boronic acid derivatives featuring alkynes and dyes, enables efficient click bioconjugation and cell imaging applications. Such features have potential to establish the method as an invaluable asset in protein labelling (Figure 2b).

Continuing in this trend, Yudin *et al*.^[19]^ identified the crucial role of the hydrogen bond between endocyclic amines and adjacent amide NH groups in achieving conformational homogeneity in peptide macrocycles. They also employed *N*‐terminal proline and modified glutamic acid to initially introduce an alkenyl boronic acid. This was followed by employing the Petasis reaction with glyoxylic acid, facilitating macrocyclisation (Figure 2b). The reaction yields diverse macrocycles (**9**) with unique features like endocyclic reduced amides and exocyclic carboxylic acids, alongside alkenyl or (hetero)aryl backbone linkers. The conformational rigidity of these macrocycles, evident in their NMR spectra, highlights their potential in modulating protein‐protein interactions, important for the development of bioactive compounds and drug discovery.

As is apparent from the lysine and proline examples, the Petasis reaction seems to work well in the presence of *sp* ^*3*^ hybridised amines. Interestingly, the reactivity of similarly nucleophilic *sp* ^*2*^ amines (such as arginine, histidine, and tryptophan), that occur in peptides as well, have been elucidated only very recently *via* the TMPR concept (**11**, Figure 2c). Krajcovicova and Spring observed an excellent chemoselectivity in the Petasis reaction in a competition experiment for tryptophan's indole moiety versus histidine's imidazole and the protected guanidine moiety of arginine. The further application of the TMPR method with incorporated arylboronic acid *via* a modified lysine, yielded a diverse set of short macrocyclic peptides at the *i,i*+4 and *i,i*+7 positions (see Figure 4b) and allowed for an easy and rapid late‐stage functionalisation with different tags from one common peptide intermediate, without a need to resynthesise it from the scratch.^[10]^

## Peptide Functionalisation of Tryptophan Using Transition Metal Catalysis

Metal‐catalysed C−H functionalisation is a particularly useful strategy that enables selective modification of tryptophan's indole core. C−H functionalisation has emerged in recent years as a transformative platform in organic synthesis and has shown great promise in the functionalisation of peptides with expanded structural diversity. The careful selection of a transition metal catalyst plays a pivotal role in C−H activation reactions, influencing both efficiency and selectivity in the transformation process.

Rhodium catalysts are renowned for their ability to activate and facilitate diverse C−H functionalisation reactions. Demonstrating high reactivity, they excel in promoting challenging transformations under mild reaction conditions.

**Figure 3:**
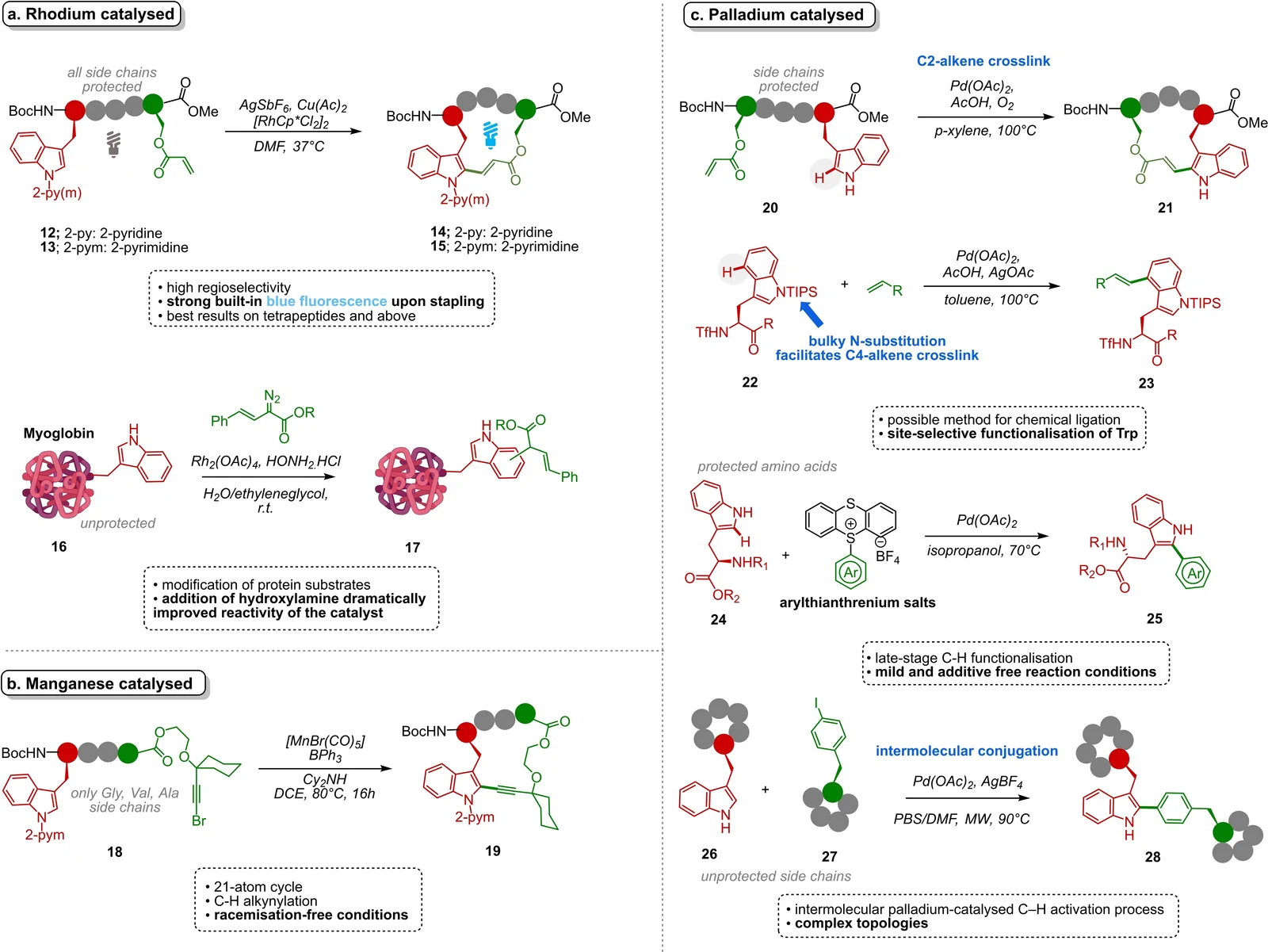
Transition metal methods.[^2^, ^5^, ^6^, ^7^, ^8^, ^24^]

The incorporation of tryptophan, coupled with a late‐stage C(*sp* ^2^)−H olefination strategy, has empowered the synthesis of stapled peptides **14**–**15** featuring integrated fluorescence and macrocyclic structures (Figure 3a).^[24]^ Employing this strategy yielded stapled peptides exhibiting enhanced stability against proteolytic degradation and a robust binding affinity for integrin αvβ3 in cell imaging experiments. The strategic placement of pyridine/pyrimidine groups on the *N* ^1^‐position of Trp served as directing groups, showcasing excellent regioselectivity and efficiency in Rh‐catalysed functionalisation, with [RhCp*Cl_2_]_2_ identified as the most suitable catalyst.^[24]^

This augmentation increased the reactivity and chemoselectivity of the reaction, leading to the formation of olefinated tryptophan derivatives characterised by strong blue fluorescence. Extending this methodology to dipeptides, tripeptides, tetrapeptides, pentapeptides, and hexapeptides resulted in the creation of stapled peptides with diverse ring sizes. The stapling reaction proved highly efficient for tetrapeptides and above, yielding stapled peptides with good yields (45–68 %).

Another highly selective method for modifying tryptophan residues using rhodium catalysis in aqueous solution was developed by Francis *et al*. (Figure 3a).^[7]^ Protein substrates can be modified at low concentrations and showcase excellent selectivity. Testing on myoglobin, it resulted in a clean conversion to modified products (**17**). Peptide analysis confirmed site selectivity, indicating modification at specific tryptophan residues.

Aimed to develop a method for an efficient peptide modification accomplished by less‐toxic manganese catalysts, Ackermann *et al*.^[2]^ sought to expand the substrate scope and enable step‐economical C−H functionalisation with various haloalkynes, including those with fluorescent labels, steroids, and amino acids (Figure 3b). The manganese‐catalysed C−H alkynylation allowed for efficient late‐stage diversification of acyclic peptides under racemisation‐free conditions, which is highly sought after. The method also facilitated the assembly of macrocycles, as demonstrated by the successful synthesis of a 21‐membered cyclic peptide **19**. It is of note that the same group successfully optimised C−H alkylation on tryptophan residues using ruthenium^[3]^ and cobalt^[4]^ catalysts as well.

Palladium, however, is still by far the favourite catalyst in tryptophan‐mediated transformations. Wang *et al*.^[8]^ aimed to develop a method for late‐stage peptide macrocyclisation using palladium‐catalysed C−H olefination of tryptophan residues (Figure 3c). Their motivation stemmed from the potential of transition‐metal‐catalysed C−H activation to enhance the structural diversity of peptides. The key highlights of their work include the utilisation of the peptide backbone as endogenous directing groups and the achievement of site‐selective C(*sp* ^2^)−H olefination at the *C* ^2^ and *C* ^4^ positions of tryptophan residues (**21** vs **23**). Macrocyclisation of the peptide by Trp(*C* ^2^)‐alkene crosslinks led to constrained and compact peptide structures with significantly enhanced cell permeability (Figure 3c).

Creating unique constrained peptides with a covalent bond between tryptophan and phenylalanine or tyrosine residues has been a motivation for Albericio *et al*.^[6]^ Their aim was to address the limited therapeutic profile of natural peptides due to their conformational freedom and metabolic instability. The preparation of such peptides was achieved in solution and on solid‐phase directly from the corresponding sequences having an iodo‐phenylalanine through an intermolecular palladium‐catalysed C−H activation process (Figure 3c).

Using arylthianthrenium salts, Ackermann *et al*. attempted to late‐stage functionalise tryptophan residues in short peptides (**25**) by palladium catalysis (Figure 3c), which proven to be especially useful in attaching different drugs such as clofibrate and gemfibrozil.^[5]^

All of these methods are efficient in peptide modification, ligation, and generation of peptide macrocycles. With rational design, these C−H activation methods hold great potential in generating complex peptide macrocycles, that often display enhanced cellular permeability. However, with the recent focus on greener synthetic approaches in the pharmaceutical industry, the field is slowly shifting more towards the metal‐free methods which have indisputable advantages especially by using more environmentally benign reagents.

## Peptide Functionalisation of Tryptophan Using Transition Metal‐Free Methods

The use of tryptophan for peptide stapling using non‐transition metals approaches is significantly less prevalent, which was also one of the key aspects that inspired and catalysed the development of the TMPR concept. The crucial advancements in this area have been done by Johannes *et al*.,^[12]^ using aldehyde‐bridged intramolecular condensation, and Taylor *et al*.,^[14]^ applying photochemistry.

**Figure 4:**
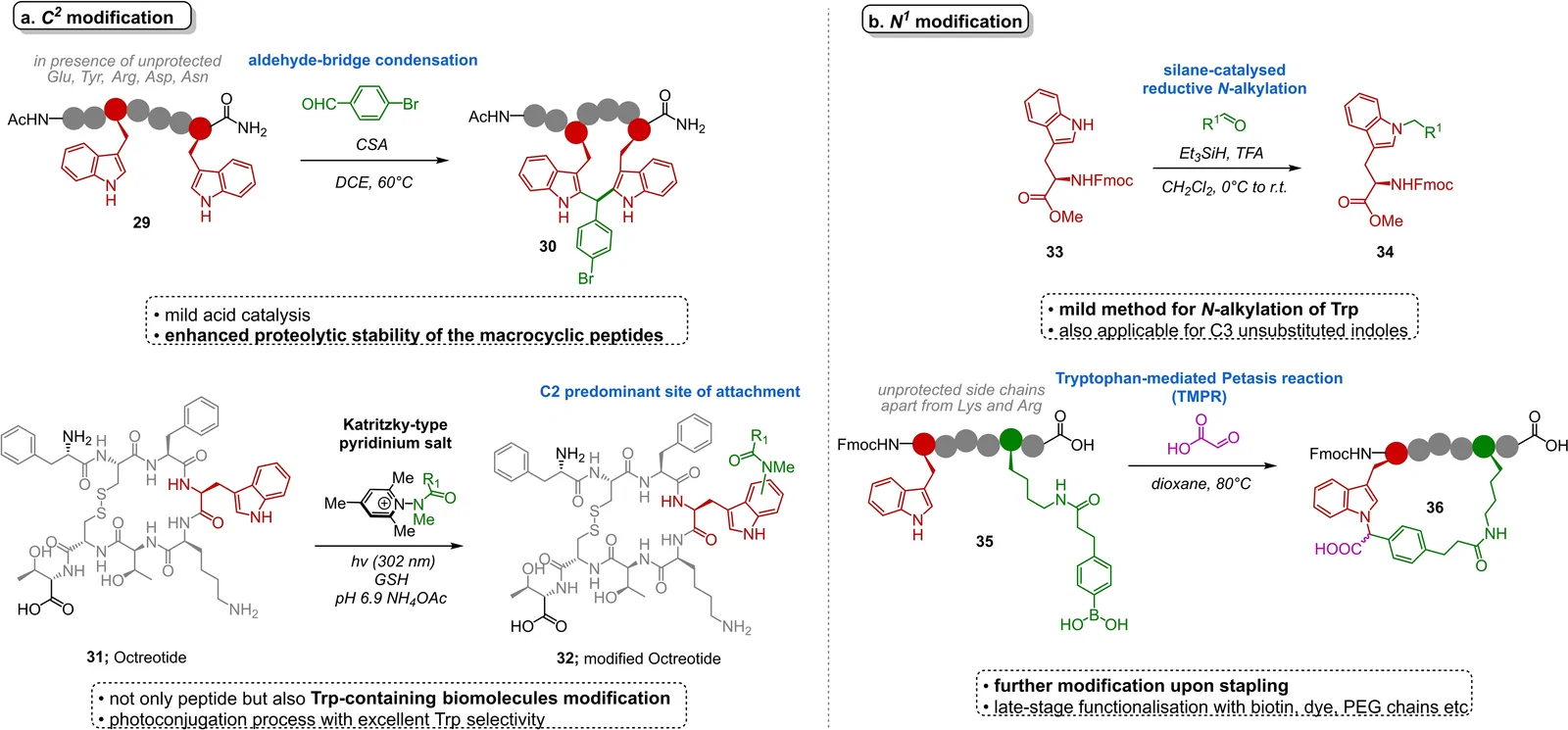
Transition metal‐free methods.[^10^, ^11^, ^12^, ^14^]

The aldehyde‐bridged condensation involves linking two tryptophan residues (**29**) at the *C* ^2^ position of the indole moieties *via* acid‐mediated condensation with an aldehyde (Figure 4a). Using skatole as a model substrate to mimic tryptophan, condensation reactions were then carried out in the presence of 4‐bromobenzaldehyde and an acid. A key aspect of this method is the use of camphor sulphonic acid (CSA) in the condensation process, yielding the stapled peptide **30** (Figure 4a). This approach not only enhances the peptide‘s stability against proteolytic cleavage but also shows promise for developing systemic peptide‐drugs resistant to proteolytic degradation. The latter described a photochemical process for selective modification of tryptophan in Octreotide **31**, employing an electron‐responsive Katritzky‐type *N‐*substituted pyridinium salt (*N*‐carbamoylpyridinium salt). Initiating a photoinduced electron transfer (PET) process at 302 nm between Trp and the pyridinium salt, this reaction is characterised by its excellent site selectivity for Trp and its tolerance to other redox‐active amino‐acid residues, such as Tyr, His or Met. Remarkably, it operates efficiently in pure aqueous conditions, without the need for organic co‐solvents or photocatalysts, and can label biomolecules of various sizes (Figure 4a).

From the described methods (both metal‐ and non‐metal catalysed) it is apparent that the synthetic approaches to selectively modify tryptophan targeted mainly the *C* ^2^‐position of the indole core. However, the moderate nucleophilicity of the *N* ^1^‐position opens doors for other synthetic transformations, such as reductive amination or multicomponent reactions. Therefore, the TMPR concept focused solely on a utilisation of *N* ^1^‐position of Trp, which has not been seen much before. As one of the very few examples of *N* ^*1*^‐alkylation could serve the work of DelMonte and Frantz *et al*.^[11]^ where acid catalysed silane‐mediated reductive *N* ^*1*^‐alkylation was applied (Figure 4b). The application of the multicomponent Petasis reaction by Krajcovicova and Spring on deprotected tryptophan yielded *N* ^1^‐substituted derivatives and enabled synthesis of both stapled and labelled peptides (**36**, Figure 4b), including water soluble PEG chains, biotin or dye tags. This makes the concept versatile for various other late‐stage functionalisations *via* a simple amide coupling reaction due to the harnessing of a protected peptide that would not cause cross‐reactivity with the other peptide side chains. This simplifies the preparation of compound libraries and suggests broader applications in peptide modification. Together, these studies highlight the evolving role of tryptophan in peptide chemistry, offering novel methods for enhancing peptide stability, functionality, and therapeutic potential.

## Summary and Outlook

The key publications presented here collectively summarise key synthetic advancements focusing on tryptophan's role in peptide stapling and the Petasis reaction in labelling and stapling of peptides. The discussed approaches offer high chemoselectivity and stereoselectivity, enabling the introduction of functional groups in a controlled manner. The development of constrained peptides through these methods has led to the improvement of pharmacokinetic properties, including stability and cell permeability. Notably, the creation of new topological architectures in peptides addresses the challenges in targeting protein‐protein interactions and other complex biological pathways. These methods not only enhance the therapeutic potential of peptides but also simplify the synthesis of complex peptide‐based compounds. Looking forward, the field could explore further the integration of these advanced methods for protein and antibody modifications, especially in areas like drug delivery and the development of therapeutics targeting challenging protein‐protein interactions. The use of tryptophan in Petasis reactions, as demonstrated in the TMPR concept, is particularly promising for its synthetic versatility and the potential for rapid late‐stage functionalisation, which seemed to be challenging in the past. The advancement in multicomponent reactions, including the development of more controlled and versatile methods like the TMPR reaction, indicates a trend towards more precise and efficient peptide modifications. This progress is expected to lead to the development of new peptide‐based therapeutics with enhanced stability, specificity, and efficacy. Future research will likely continue to expand the scope of substrates and functional groups, exploring the full potential of these innovative peptide modification strategies in chemical biology.

## Conflict of Interests

The authors declare no conflict of interest.

## Biographical Information

*Sona Krajcovicova was born in Bratislava, Slovakia. She obtained her Ph.D. in Organic Chemistry in 2019 at Palacky University Olomouc, Czech Republic under a guidance of Assoc. Prof. Miroslav Soural, focusing on novel high‐throughput conjugation methods of drug‐like molecules for chemical biology. Awarded by two prestigious national postdoctoral fellowships from the Experientia Foundation and Czech Science Foundation in 2021 and 2022 to conduct her research abroad, she joined Prof. David Spring's group at the University of Cambridge, UK, where she now focuses on developing innovative synthetic approaches for next‐generation therapeutics, with a special interest in stapled peptides and antibody‐drug conjugates (ADCs)*.